# Association of serum leptin at 24–28 weeks gestation with initiation and progression of labor in women

**DOI:** 10.1038/s41598-022-19868-0

**Published:** 2022-09-26

**Authors:** Ki’ara K. R. Branham, Elizabeth Sherman, Mojgan Golzy, Erma Z. Drobnis, Laura C. Schulz

**Affiliations:** 1 Department of Obstetrics, Gynecology and Women’s Health, University of Missouri, 1 Hospital Drive, Columbia, MO 65212 USA; 2 Family and Community Medicine, University of Missouri, Columbia, MO USA

**Keywords:** Reproductive biology, Reproductive signs and symptoms

## Abstract

Concentrations of the hormone leptin, which is produced by adipose tissue, increase with increasing BMI, whereas leptin sensitivity often declines with higher BMI. Thus, altered leptin signaling may play a role in reproductive health risks observed with increasing BMI, which include later onset and slow progression of labor. Conflicting evidence from clinical, animal and in vitro studies have suggested that leptin either promotes or inhibits labor. We hypothesized that serum leptin concentrations or serum leptin: body mass index (BMI) ratios in women may be associated with the initiation and progression of labor. Following informed consent, serum samples were collected from 90 women with singleton pregnancies at the time of routine glucose-challenge testing, for measurement of leptin. The potential influence of leptin on gestation length and cervical dilation timing were examined by multiple linear regression. Data were analyzed from 63 participants who met exclusion and inclusion criteria. Leptin concentrations (log-transformed) at 24–28 weeks gestation were not significantly correlated with first trimester BMI . Log serum leptin and leptin: BMI ratio each were significantly associated with shorter total gestation length in uncomplicated, term pregnancies. In contrast, the mid-pregnancy leptin concentrations were not associated with progression of labor, assessed by cervical dilation over time. The association between higher serum leptin and shorter gestation length is consistent with the hypothesis that leptin promotes, or is permissive for, the onset of labor.

## Introduction

The hormone leptin is produced by adipose tissue in proportion to overall fat mass and it serves as a signal to the reproductive system of adequate nutritional status, acting as a permissive factor for puberty, cyclicity, and pregnancy^1,2^. Circulating leptin levels increase with increasing body weight in men, and in both pre- and post- menopausal women^3^. Thus, some individuals with higher adipose mass are chronically exposed to high leptin levels and become resistant to this hormone^4^. Even the placenta can become leptin resistant in women with high BMI. For example, leptin receptor expression and leptin-stimulated amino acid transport were significantly reduced in placentas from women with BMI ≥ 30 kg/m^2^ vs. those from women with BMI 18.5–24.9 kg/m^2^^4,5^. As a result, pregnant women with high BMI have high circulating leptin levels, but may have reduced leptin signaling. These alterations in leptin concentration and function at high BMI suggest that leptin could play a role in reproductive health risks observed at high BMI.

Over a third of women of childbearing age in the United States have a body mass index (BMI) > 30, and they are at increased risk for slow progression of labor, as well as later onset of labor or longer overall gestation length^6–8^. Women with a BMI > 30 kg/m^2^ are at significantly greater risk of having gestation extended past 41–42 weeks^6,9,10^. The duration of labor is also extended with increasing BMI in several reports^11,12^. For example, in a study of 612 nulliparous women, the median cervical dilation time from 4 to 10 cm for those with BMI > 29 was significantly longer at 7.9 h versus just 6.2 h in those with a BMI ≤ 26 and 7.5 h for those with a BMI 26.1–29^13^ and Bishop Scores for cervical ripening were lower upon admission for women with BMI of 30 or greater^12^. Sensitivity to prostaglandins, which promote cervical ripening, is also reduced in women with BMI > 30^14^The incidence of intra partum caesarean section for labor dystocia is also higher at higher BMI^15–17^, Although uterine contractility is reduced^18^, and even differences in the maternal serum metabolome^7^ have been observed, the physiological mechanisms that underlie delays in the onset and progression of labor with increasing BMI remain poorly understood. At the same time, the incidence of preterm birth is also higher in women with BMI > 30. The relationship between BMI and preterm birth is U-shaped, with the increasing risk of preterm birth for BMIs above or below the 18–22 range, highlighting the complicated relationship between BMI and initiation of labor^19–21^.

There is limited, sometimes contradictory, evidence for a role of leptin in the onset and/or progression of labor. Evidence that leptin promotes the onset of labor includes a study in thethe Lep^*ob/ob*^ mouse, which cannot produce leptin, and is infertile. When leptin is given to the Lep^*ob/ob*^ mouse to restore estrous cycles and initiate pregnancy but then withdrawn prior to term, gestation and labor are prolonged^22^. Similarly, when leptin receptor is knocked out in progesterone receptor-expressing tissues, the average time to deliver each pup is lengthened, a greater number of stillborn pups are observed, and there is increased risk of dystocia^23^ Consistent with these observations, leptin injections promote cervical softening in the non-pregnant, hormone-primed rat^24^. Collectively, these studies suggest that leptin ensures that delivery can progress at the appropriate time, and raise the possibility that greater leptin signaling could hasten delivery.

However, evidence from other studies suggests that leptin may delay or slow labor, or have no effect. In mice with just one fully functional copy of the leptin receptor gene, myometrial contractions are not different than those in control mice, although the underlying signaling changes^25^. In samples from both women and rodent models, incubation of myometrial strips with leptin inhibits contractility, although it should be noted that inhibition occurs at superphysiological concentrations of 1 µM^26–29^. Cord blood leptin concentrations, reflecting fetal rather than maternal leptin, are positively associated with longer duration of labor^30,31^. Mid-gestation leptin levels were also significantly higher in women with failed induction of labor in one study, but after adjustment for BMI, were not associated with failed first stage of labor in another^32,33^. Thus, the question of whether leptin modulates gestation length or progression of labor remains open.

The goal of the present study is to test whether maternal serum leptin concentrations, measured in mid-pregnancy, are significantly associated with the onset of labor, as measured by gestation length, or with the progression of labor, measured by the timing of cervical dilation.

## Methods

### Patient population

The study was approved by the University of Missouri Health Sciences Institutional Review Board, and all participants provided written informed consent. It was conducted in accordance with all relevant regulations for Human Subjects research, and the Declaration of Helsinki. Women ≥ 18 years of age bearing a singleton pregnancy were recruited from the University of Missouri Women’s and Children’s Hospital, Smiley Lane Clinic, and Missouri OB/GYN Associates in Columbia, Missouri at a regularly scheduled, second trimester obstetric visit prior to oral glucose challenge testing.

Participant data were included in analysis if a body mass index had been recorded in the electronic medical record during the first trimester of pregnancy. Criteria for exclusion from analysis included those conditions that might modify the labor process or shorten gestation length: multiple gestation pregnancy, gestational hypertension, pre-eclampsia, preterm premature rupture of membranes (PPROM), premature rupture of membranes, scheduled or planned cesarean delivery, or fetal malpresentation. Additionally, those who underwent induction of labor by administration of oxytocin (Pitocin), prostaglandin, or manual rupture of membranes for post-term pregnancy as documented in the medical record were excluded from analysis, as these inductions would alter the length of gestation and could affect the rate of cervical dilation^34,35^. Routine administration of Pitocin to augment uterine contractions was not excluded, but was accounted for in the statistical analysis of cervical dilation timing.

Data on fetal sex, parity, induction of labor, labor augmentation, maternal age, first trimester BMI, gestational weight gain and cervical dilation timing were collected from the electronic medical record.

### Serum leptin determination

During routine glucose challenge testing at 24–28 weeks gestation, following overnight fast, an additional blood sample was taken for leptin measurement at 1 h post glucose challenge. The samples were centrifuged for 15 min at 1000 × g for serum separation, and then serum was stored at -80 °C until use. The Human Leptin Quantikine ELISA kit (R&D Systems) was used according to manufacturer’s protocol. Serum samples were diluted 100-fold to fit within the assay range. Briefly, both serum samples and leptin standards were diluted, and added to the coated microplate. After washing, wells were incubated with human leptin conjugate solution for 1 h, and then washed before incubating with substrate solution for an additional 30 min. Results were read on a Biotek plate spectrophotometer (Winooski, VT). To validate the assay, serial dilutions of serum were used to confirm linearity, and known quantities of the leptin standard solution provided with the kit were added to serum samples to assess recovery. The intraassay and interassay coefficients of variation (CV%) were 2.5% and 7.1%, respectively.

### Statistical analysis

A sample size calculation was performed for multiple regression analysis by using G*Power Software^36^. For a power of 0.8, alpha = 0.05, it predicted that 68 subjects would be needed to detect an effect size, F^2^, of 0.15 on gestation length, the primary outcome, in a regression model with two predictors, where both predictors and dependent variables are continuous. The effect size was calculated by using Cohen’s F^2^, where F^2^ = R^2^/(1-R^2^)^37^, with R^2^ estimated from the impact of BMI on gestation length in multiple regression analyses^6,38,39^. Additionally, Cohen defines 0.15 as a “medium” effect size^37^. It was estimated that approximately 25% of patients recruited in mid-pregnancy would be lost to follow-up or develop one of the criteria for exclusion from analysis prior to study completion. Therefore, a goal of recruiting 90 participants was established.

Descriptive statistics were used to summarize patient data using frequency and percentages for categorical variables, and mean ± standard deviation (SD) and the median with interquartile range (IQR) for continuous variables. A linear regression was used to assess the relationship between BMI and serum leptin. As leptin concentrations and leptin:BMI ratios were not normally distributed, these variables were log-transformed to achieve normal distributions. Separate generalized linear regression models were used to assess the effect of either log (leptin) or log (leptin:BMI) on gestation length, controlling for the following confounding variables: 1st trimester BMI, maternal age, gestational weight gain, fetal sex, Pitocin administration and parity. Stepwise backward selection was used to sequentially eliminate variables with the largest p-values to obtain the final regression models.

For analysis of progression of labor, the time at which full effacement or 10 cm cervical dilation was noted in the medical record was considered time 0 for each patient, and each prior recorded observation of cervical dilation was expressed relative to time 0^40^. A generalized estimating equations model (GEE) was used to fit a quadratic model describing the cervical dilation for all subjects, taking repeated measures within subject into account.

All tests were two-sided, with a level of significance 0.05. SAS 9.4^®^ software was used for statistical analysis of data.

### Ethics approval and consent to participate

The study was approved by the University of Missouri Health Sciences Institutional Review Board, and all participants provided written informed consent.

## Results

### Data stratification

**Figure 1:**
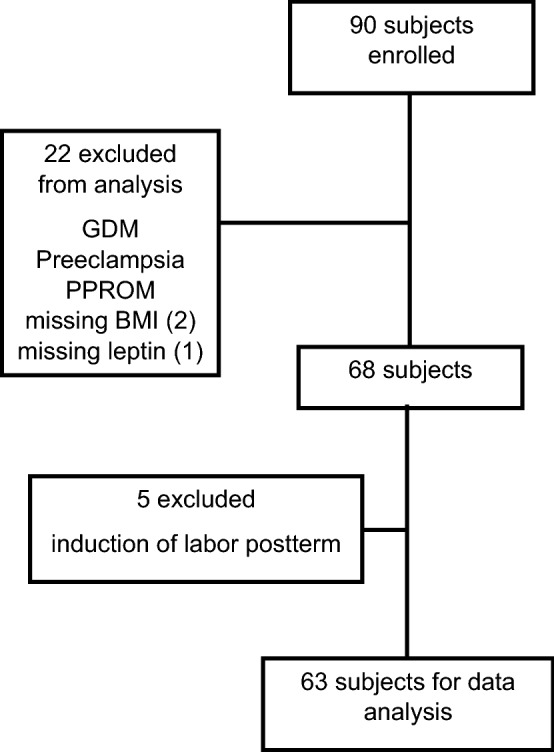
Number of participants enrolled and included in analysis. GDM = gestational diabetes mellitus. PPROM = preterm premature rupture of membranes.

Ninety subjects were enrolled and data from nineteen of them were excluded from analysis due to complications considered exclusion criteria, as listed in *Materials and Methods* (Fig. 1). Leptin data were missing for one subject and BMI values were missing for two additional subjects. Additionally, data from five participants were excluded from analysis due to induction of labor for post-term pregnancy. Data from the remaining 63 subjects were analyzed (Fig. 1). Patient characteristics are displayed in Table 1 for continuous variables, and Table 2 for categorical variables. The mean length of gestation, 275.9 days, is equivalent to 39.4 weeks.

**Table 1:** Patient characteristics for continuous variables (N = 63).

Variable	Mean ± SD	Median	Interquartile range
Maternal age (years)	27.8 ± 5.0	28	24–31
1st trimester body mass index (kg/m^2^)	27.0 ± 5.7	25	23–31
Gestational weight gain (kg)	11.3 ± 6.0	11.5	8.4–15.7
Leptin (ng/mL)	43.9 ± 47.2	32.6	21.2–45.6
Leptin: BMI ratio	1.66 ± 1.9	1.15	0.69–1.7
Length of gestation (days)	275.9 ± 5.2	275	273–279

**Table 2:** Patient characteristics, categorical variables.

Variable	Frequency (%)	Cumulative frequency (%)
**Parity**		
0	32 (50.8%)	32 (50.8%)
1	21 (33.3%)	53 (84.1%)
≥ 2	10 (15.9%)	63 (100.0%)
**Fetal sex**		
Male	37 (58.7%)	37 (58.7%)
Female	26 (41.3%)	63 (100.0%)
**Pitocin**		
Yes	34 (54%)	34 (54%)
No	29 (46%)	63 (100.0%)
**BMI category**		
< 25	28 (44.4%)	28 (44.4%)
25–29.9	16 (25.4%)	45 (69.8%)
≥ 30	19 (30.2%)	63 (100.0%)

### Relationship between first-trimester BMI and mid-gestation leptin concentrations

**Figure 2:**
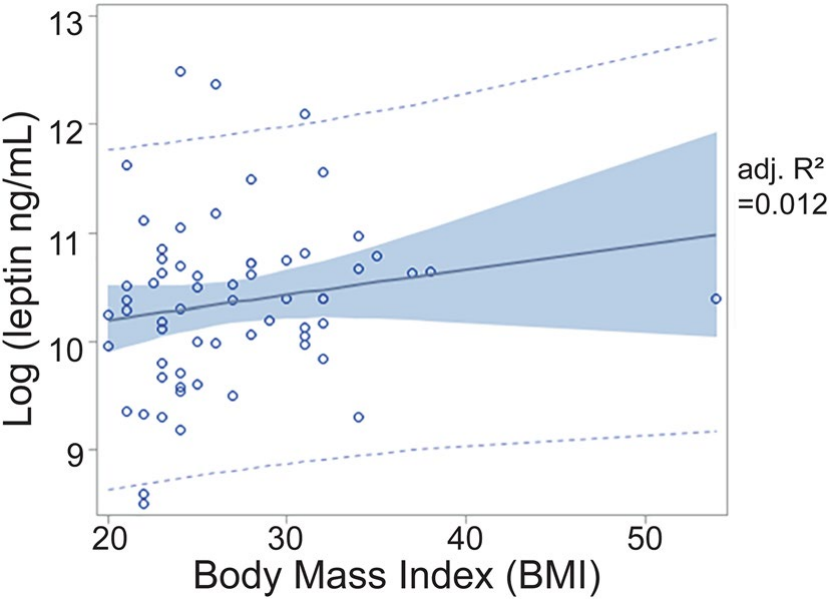
Relationship between body mass index (BMI) in the first trimester and mid-gestation serum leptin concentrations. Leptin concentrations are log-transformed to achieve a normal distribution. There is a weak relationship (β = 0.02, *p* = 0.18). Each circle represents a single observation, the solid line is the best fit line, and the shaded area indicates the 95% confidence interval.

The mean leptin concentration at 24–28 weeks gestation for all participants was 43.9 ± 47.2 ng/mL. Serum leptin was not significantly dependent upon first trimester BMI (Fig. 2; β = 0.02, *p* = 0.18). When BMI was stratified by category, there were no significant differences in leptin concentration amongst those with a BMI less than 25 kg/m^2^, a BMI between 25 and 29.9 kg/m^2^, and a BMI of 30 kg/m^2^and higher (F-value = 1.85, *p* = 0.16).

### Relationship between mid-gestation leptin concentrations and length of gestation

**Figure 3:**
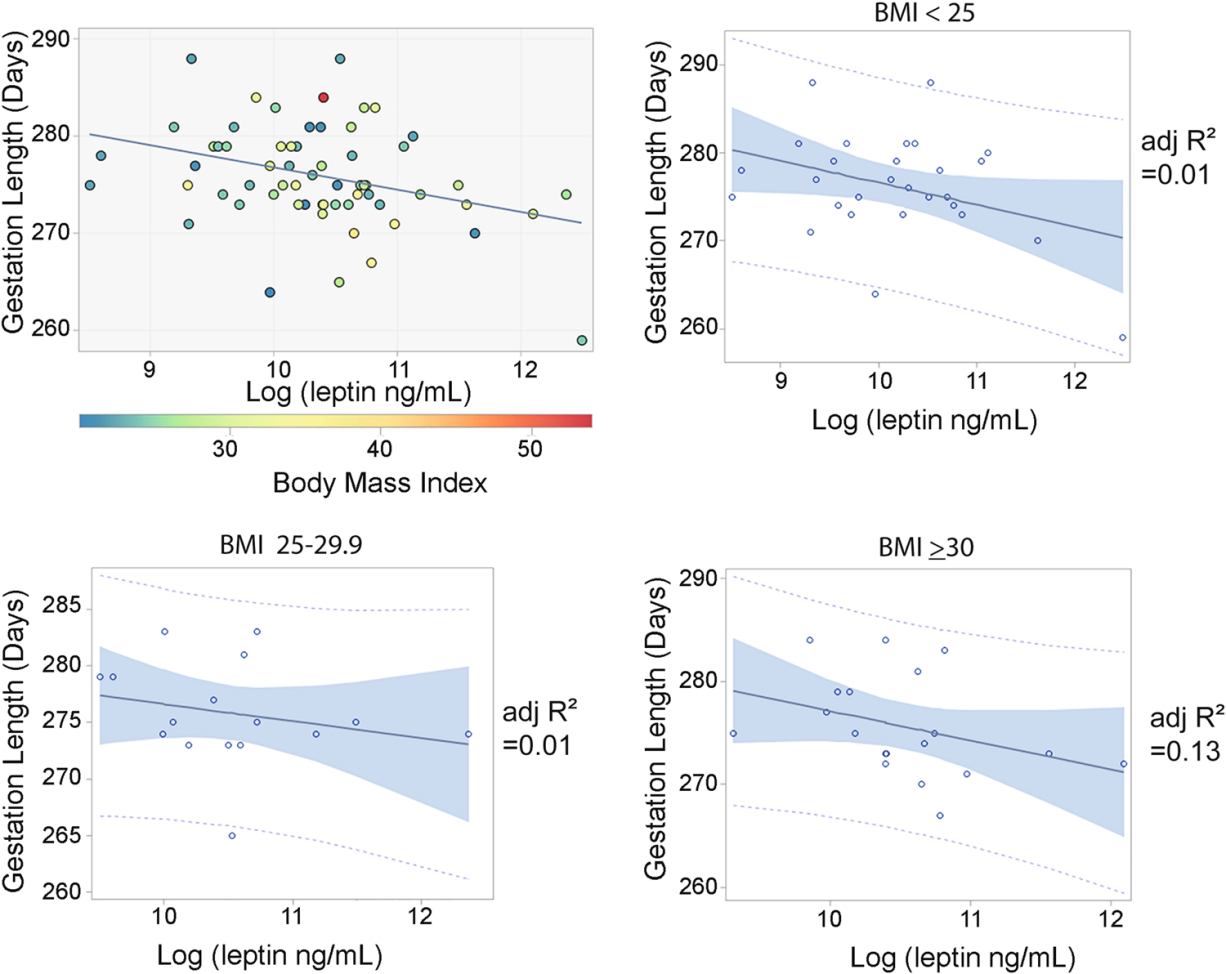
(**a**) Relationship between mid-gestation serum leptin concentrations and total gestation length in days. Leptin concentrations are log-transformed to achieve a normal distribution. For each individual, BMI is indicated by marker color. Gestation length is significantly related to log leptin (*p* = 0.01) (**b**) Relationship between serum leptin concentrations and length of gestation for individuals with BMI < 25 (**c**) Relationship between serum leptin concentrations and length of gestation for individuals with BMI 25–29.9 (**d**) Relationship between serum leptin concentrations and length of gestation for individuals with BMI > 30. Each circle represents a single observation, the solid line is the linear regression, and the shaded area indicates the 95% confidence interval.

In generalized linear regression analysis, including the variables maternal age, 1st trimester BMI, gestational weight gain, fetal sex, Pitocin administration and parity, only serum leptin concentrations showed a significant, negative correlation with gestation length (*p* = 0.013, model *p* = 0.26). Backward selection was performed to remove the non-signficant variables leaving a significant final regression model that included log leptin, gestation weight gain and fetal sex, with log leptin as the most significant predictor (Table 3, Fig. 3a). In a subgroup analysis, the relationship between serum leptin concentration and gestation length was visualized within each BMI category (Fig. 3b–d). Although the sample size of each subcategory was not large enough to draw conclusions, the relationship between leptin and gestation length did not appear to differ between categories.

**Table 3:** Final generalized linear model assessing the effect of log leptin and potentially confounding variables on length of gestation after stepwise backward selection.

Model	N = 63	R^2^ = 0.128	*p* = 0.043
Parameter	Estimate	95% CI	*p*-value
Gestational weight gain (kg)	0.10	− 0.12–0.32	0.44
Log [leptin (ng/mL)]	− 2.21	− 4.90 to − 0.53	**0.0071**
Fetal sex (female vs male)	0.70	− 1.98–3.38	0.60

Significant values are in bold.

**Figure 4:**
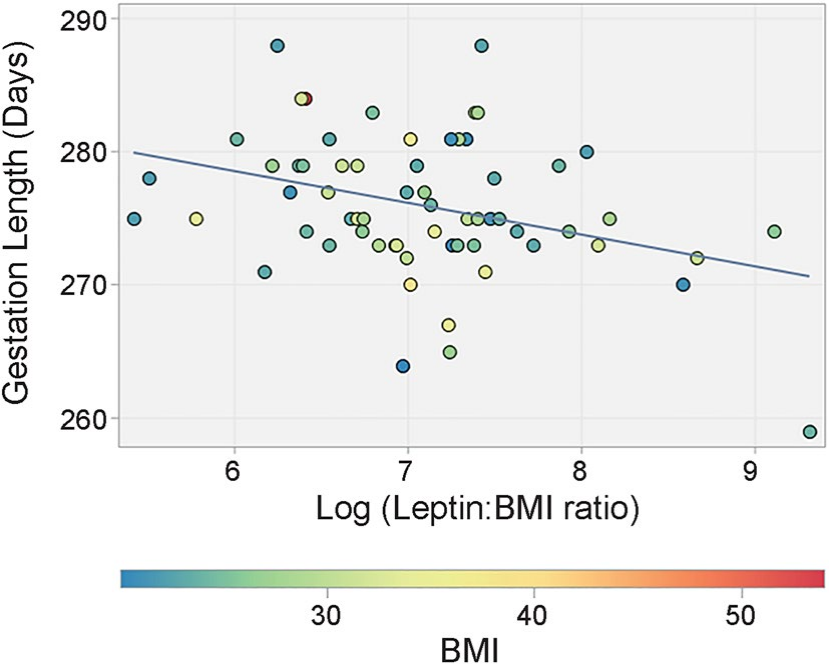
Relationship between mid-gestation serum leptin concentrations, adjusted for BMI, and total gestation length in days. The leptin:BMI ratio is log-transformed to achieve a normal distribution. For each individual, BMI is indicated by marker color.

A generalized regression model was also constructed using leptin:BMI ratio, or BMI-adjusted leptin concentrations, maternal age, BMI, gestational weight gain, fetal sex, oxytocin and parity as predictors of length of gestation (*p* = 0.27). After backward selection, a final regression model with log leptin:BMI ratio, gestational weight gain and fetal sex as variables showed a significant relationship with gestation length (Table 4, Fig. 4).

**Table 4:** Final generalized linear model assessing the effect of log leptin:BMI ratio and potentially confounding variables on length of gestation after stepwise backward selection.

Model	N = 63	R^2^ = 0.139	*p* = 0.031
Parameter	Estimate	95% CI	*p*-value
Gestational Weight Gain (kg)	0.12	− 0.10–0.33	0.44
Log [Leptin (ng/mL): BMI (kg/m^2^)]	− 2.37	− 4.07 to − 0.66	**0.0048**
Fetal Sex (Female vs Male)	0.73	− 1.92–3.39	0.58

Significant values are in bold.

### Relationship between serum leptin concentrations and progression of labor

**Figure 5:**
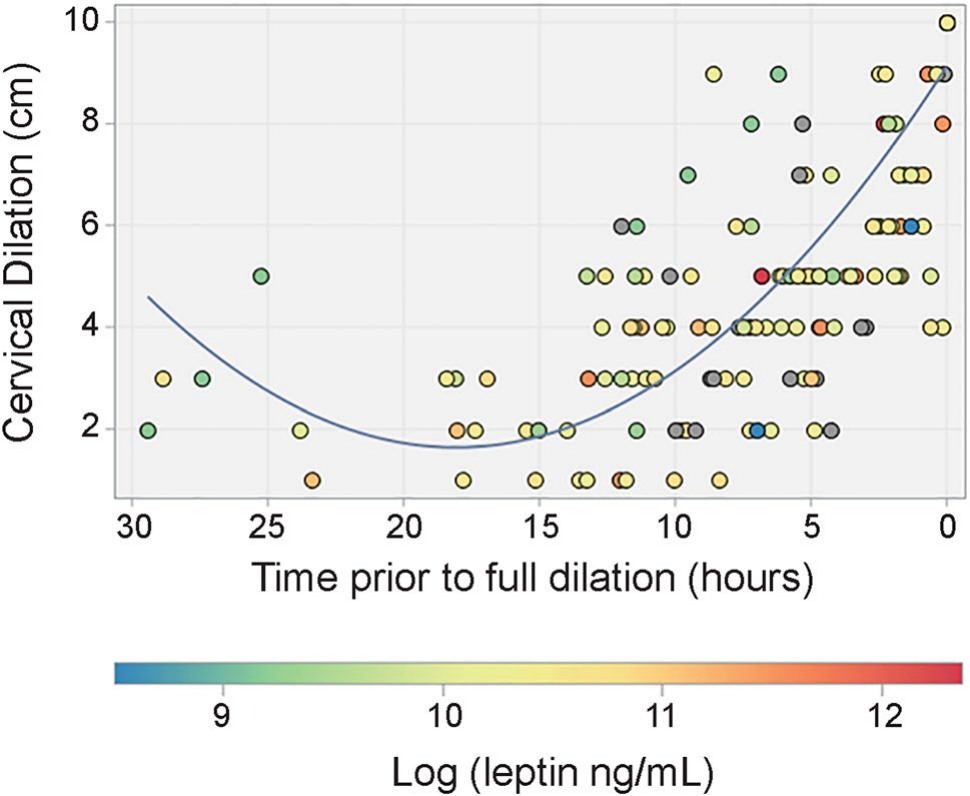
The timing of cervical dilation across all subjects. Time 0 represents the time at which full dilation or 10 cm was achieved. Each marker represents one observation of cervical dilation. The color of each marker indicates log (leptin). Leptin was not a significant factor in timing of cervical dilation (β = −0.06, *p* = 0.7) when controlling for confounding variables.

A generalized estimation model was used to assess the relationship between serum leptin concentration and the progression of labor, controlling for confounding variables (Fig. 5, Table 5). Leptin was not a significant determinant of the labor curve, nor was there any significant relationship with maternal age, gestational weight gain, or oxytocin administration. While the relationships were not statistically significant, there was some indication that BMI, fetal sex and parity influenced cervical dilation.

**Table 5:** Generalized estimating equations model assessing the effect of log of leptin and potential confounding variables on cervical dilation over time.

Model	N = 63		
Parameter	Estimate	95% CI	*p*-value
Cervical dilation time	−0.823	−0.939 to −0.708	< .0001
Cervical dilation time^2^	0.021	0.015–0.028	< .0001
Maternal age	−0.025	−0.092–0.042	0.46
1st trimester body mass index (kg/m^2^)	−0.024	−0.054–0.006	0.12
Gestational weight gain (kg)	−0.009	−0.044–0.026	0.61
Log leptin (ng/mL)	−0.058	−0.401–0.284	0.74
Fetal sex (female vs male)	0.460	−0.127–1.046	0.12
Pitocin (no vs yes)	−0.014	−0.591–0.562	0.96
**Parity**			
0 vs ≥ 2	0.477	−0.264–1.218	0.21
1 vs ≥ 2	−0.380	−1.01–0.246	0.23

## Discussion

The major finding of this study is that serum leptin concentrations at 24–28 weeks, especially BMI-adjusted leptin concentrations, are negatively associated with gestation length. This suggests that leptin may have a role in promoting the initiation of labor. In contrast, no relationship was observed between serum leptin and cervical dilation time.

Amongst the 63 subjects in this study, there was only a weak relationship between serum leptin concentrations at 24–28 weeks gestation and BMI recorded in the first trimester. It is possible that leptin concentrations were somehow altered by the glucose challenge testing, as insulin regulates leptin production, but only chronic fasting and not acute feeding has been shown to alter serum leptin^41^. Numerous studies in non-pregnant humans and animal models show a tight correlation between serum leptin concentrations and weight, BMI or adiposity^42^. However, this relationship is weakened or absent in pregnancy^43,44^, though in several studies a significant relationship remains^45–47^. The weakened relationship is not surprising, as the placenta becomes a major source of leptin production during pregnancy, and its production is partially hormone-regulated, whereas adipose tissue is the major leptin source outside of pregnancy^43,48–51^. Although maternal leptin is inversely associated with gestational weight gain in the first trimester, consistent with its role in suppressing appetite and weight gain, leptin is positively associated with weight gain in the second trimester, suggesting a positive feedback mechanism^52^. One important implication of the altered leptin-BMI relationship in pregnancy is that leptin:BMI ratio, sometimes used as a measure of leptin resistance, likely does not meaningfully reflect leptin resistance during pregnancy.

Increases in both serum leptin and leptin:BMI ratio were associated with shorter gestation length, with leptin:BMI ratio being the most significant predictor of gestation length of all the factors examined. The fact that raw leptin concentrations and leptin:BMI ratios changed gestation length in the same direction, and by a similar magnitude, again suggests that leptin:BMI ratio is a BMI–adjusted measure of leptin action, not a measure of leptin resistance.

The median BMI for participants in this study , at 27 ± 5.7 kg/m^2^, was just above the “normal” BMI range, and slightly lower than the U.S. average for adult women of 29.6^53^. This raised a concern that participants with a high BMI may have been disproportionally excluded from analysis because high BMI is a risk factor for events or conditions, including pre-eclampsia^54,55^, gestational hypertension^8^, and planned caesarean section^56^, for which subjects were excluded. However, when mean BMI, gestational weight gain, maternal age, parity and fetal sex were compared between the 63 subjects included in analysis and the subjects that were excluded, no significant differences were detected (Student’s t-test, Chi-square *p* > 0.4). Whatever the reason for the lower representation of high BMI subjects in this study, it would be necessary to examine more women of higher BMI to determine with high confidence whether the leptin- gestation length relationship differs with BMI, although we found no evidence for that within this sample.

Of the factors potentially influencing gestation length and cervical dilation considered here, only serum leptin and leptin:BMI ratio had any significant effects. Maternal age and fetal sex were included in the analyses of gestation length and cervical dilation times because of previous evidence that they may play a significant role. Duration of the second stage of labor increases with maternal age, with one study of nearly 32,000 births finding a 97-min increase in average second stage duration in women over 39 compared to those under 20^57–60^. Similarly, women carrying a male fetus have increased risk of failure to progress^61^ and a longer first stage of labor, although the delay (0.6 h) is modest^62^. First pregnancies have been found to be significantly longer than multiparous pregnancies^59,63^, although others have not found a significant effect of parity^60^. Like higher BMI, excessive gestational weight gain is associated with prolonged labor complications, cesarean delivery and prolonged gestation lengths^64,65^, though there are less data available. That none of these factors (maternal age, fetal sex, gestational weight gain, parity) significantly impacted gestation length in this study probably reflects the relatively small sample size, as hundreds, or even thousands of births were examined in the previous studies that did find these factors significant. This is additionally suggested by the partial evidence (p < 0.2) for an impact of BMI, fetal sex and parity on cervical dilation times. That there was no significant relationship between Pitocin administration and gestation length suggests that we were able to sufficiently discriminate between those cases in which it was given to initiate labor, which were excluded from analysis, and cases in which it was given after the natural initiation of labor, which were included.

Given the extensive evidence linking higher BMI to labor dysfunction, the lack of a significant relationship between BMI and gestation length or cervical dilation was unexpected. However, average gestation length may not be as closely related to BMI as the incidence of post-term pregnancy. For example, a study of nearly 150,000 births in Sweden found a significant effect of BMI on average gestation length but the difference between the highest and lowest categories was only 1.5 days, and at least one smaller study found no effect^6,60^. In contrast, risk of post-term pregnancy is a consistent finding, and meta-analysis showed an odds ratio of 1.75 for gestation length ≥ 42 weeks in women with BMI of at least 40^9^. As those with scheduled induction of labor were excluded from the present analysis, post-term pregnancies were largely not included, and there were too few to analyze as a separate outcome measure. The limited evidence for a relationship between BMI and cervical dilation (*p* = 0.12) again suggests that there was not a large enough sample size to make a meaningful conclusion.

This study was powered to determine the potential effect of leptin on the initiation of labor, by examining the relationship between leptin concentrations and gestation length, or the time until initiation of labor, as a primary outcome, but also examined the relationship between mid-gestation serum leptin concentrations and cervical dilation, finding no significant effect. Because dilation times were extracted from the electronic medical record, rather than recorded systematically, some patients had only one or a few dilation times recorded. Nonetheless, the lack of an effect of leptin is consistent with a previous study of 766 Swedish women in spontaneous labor, in which there was also no significant association between leptin concentrations during active labor and the duration of labor^66^.

A potential weakness of this study is that serum leptin concentrations were measured at 24–28 weeks, whereas the outcome measures occur many weeks later. This makes it more difficult to impute a potential causal or mechanistic relationship between serum leptin and the initiation or progression of labor, as leptin concentrations were not actually measured during labor, and may have changed in the intervening time. However, leptin concentrations, after increasing through the second trimester, remain fairly consistent for the rest of pregnancy^43,67^. Here, blood samples were taken at 24–28 weeks to facilitate patient participation; it was more convenient for participants to provide a second blood sample at the time of glucose challenge testing than to schedule a blood draw specifically for the study, or to undergo sampling while in labor. Additionally, the 24–28 week sampling may also be considered advantageous for its clinical implications, as leptin concentrations as early as 24–28 weeks appear to have some value in predicting gestation length.

## Conclusions

The clinical significance of leptin:BMI ratios for the prediction of gestation length is limited, as it explains a small portion of the overall variation in uncomplicated pregnancies. This is true for many other factors that have been shown to significantly relate to gestation length, consistent with the idea that regulation of the initiation of labor in women is complex and robust^68,69^. Nonetheless, together with data from mouse models, the significant relationship between serum leptin and gestation length found here supports a role for leptin in the initiation of labor, and undercuts the idea that leptin inhibition of myometrial contractions contributes significantly to longer gestation lengths. The physiological mechanisms by which leptin may shorten gestation, and even whether leptin plays a role through central or peripheral signaling have yet to be determined.

## Data Availability

The data underlying the manuscript are presented in the manuscript or are available from the corresponding author on reasonable request.

## Abbreviations

BMI: Body mass index
Lep: Leptin
SD: Standard deviation
IQR: Interquartile range
GEE: Generalized estimating equation
